# Comparative Analysis of Genetic Structure and Diversity in *Larimichthys polyactis*, *Larimichthys crocea*, and Their Reciprocal Hybrids Based on Microsatellite Loci

**DOI:** 10.3390/ani15101360

**Published:** 2025-05-08

**Authors:** Zehui Wang, Dandan Guo, Qingping Xie, Fuliang Wei, Lin Jiang, Feng Liu, Ting Ye, Bao Lou

**Affiliations:** 1 College of Marine Science and Technology, Zhejiang Ocean University, Zhoushan 316021, China; 2 Zhejiang Key Laboratory of Coastal Biological Germplasm Resources Conservation and Utilization, State Key Laboratory for Quality and Safety of Agro-Products, Institute of Hydrobiology, Zhejiang Academy of Agricultural Sciences, Hangzhou 310021, China

**Keywords:** distant hybridization, hybrid yellow croaker, genetic diversity, genetic structure

## Abstract

In this study, we compared the genetic diversity and structure of small yellow croaker (*Larimichthys polyactis*, LP), large yellow croaker (*Larimichthys crocea*, LC), and their reciprocal hybrids (LP ♀ × LC ♂ (LCP) and LC ♀ × LP ♂ (LPC)) using 14 microsatellite loci. Our results revealed that genetic diversity was highest in LCP, followed by LP and LPC, with LC exhibiting the lowest level. Additionally, among the two hybrid progenies, the number of loci in LCP deviating from Hardy–Weinberg equilibrium was lower. This suggests that LCP is a more appropriate choice as breeding material and has the potential to enhance germplasm resources. Based on the analysis of 14 microsatellite loci, we observed that both hybrid species clustered with their respective maternal parents. Specifically, LPC exhibited a closer genetic relationship to its maternal parent than LCP did. Furthermore, the majority of genes in LPC were inherited from its maternal parent (LP). In the LCP population, approximately 63% of individuals possessed gene profiles similar to those observed in LPC, while the remaining individuals displayed a mix from both parents. This study provides a strategic direction for the efficient utilization and management of novel germplasm resources in hybrid yellow croaker. Hybrid yellow croaker serves as an intermediate breeding material, playing a significant role in the genetic improvement of *Larimichthys crocea* and *Larimichthys polyactis*.

## Simple Summary

Hybrid breeding represents a promising strategy for enhancing fish germplasm resources. In this study, we examined two hybrid offspring combinations derived from small yellow croaker (*Larimichthys polyactis*) and large yellow croaker (*Larimichthys crocea*), aiming to identify superior hybrid materials suitable for aquaculture applications. Through genetic analysis, we discovered that the hybrid LCP (female large yellow croaker × male small yellow croaker) exhibited significantly higher genetic diversity and greater genetic stability compared to its reciprocal hybrid, LPC. Notably, approximately 50% of LCP individuals displayed unique genetic combinations inherited from both parental species, whereas LPC hybrids predominantly retained maternal genetic traits. These results indicate that the LCP hybrid is a valuable germplasm resource with considerable potential. Our findings provide valuable insights and practical guidance for selecting optimal hybrid lineages to enhance the genetic quality of these economically significant marine fishes.

## 1. Introduction

Small yellow croaker (*Larimichthys polyactis*, LP) and large yellow croaker (*Larimichthys crocea*, LC) are two closely related species within the genus *Larimichthys* of the family Sciaenidae. Significant differences exist between adult LP and LC in terms of body size and distribution areas. LP is predominantly distributed in the East China Sea and its northern adjacent waters, whereas LC is primarily found in the East China Sea and its southern adjacent regions. LP exhibits a shorter breeding cycle and greater tolerance to low temperatures, while LC demonstrates faster growth rates and stronger resilience to high temperatures. Both species are highly prized by consumers for their delectable flesh and play a crucial role in China’s marine fishing industry. However, during the last century, overfishing and environmental changes led to a gradual decline in population sizes of these two species, bringing their fishery resources to the brink of collapse. To better protect the wild resources of LP and LC, China implemented a series of conservation measures in the 1990s, including fishing moratoriums and maximum catch limits. Simultaneously, to meet consumer demand, both species achieved full artificial breeding by the end of the 20th century and in 2016, respectively [1,2]. This has provided a solid foundation for the selection of superior breeds and restocking programs. Studies have shown that, despite severe overfishing of wild LP and LC populations, their genetic diversity has not significantly decreased [3,4]. However, with rapid developments in aquaculture, prolonged inbreeding has led to a marked reduction in genetic diversity within cultivated populations of LP and LC, particularly in LC, where genetic diversity has decreased by 53% compared to wild populations [5,6]. Consequently, this has resulted in genetic degradation issues, such as smaller body sizes and lower survival rates, in the current cultivated populations of LP and LC [7,8].

High genetic diversity can enhance the growth rate and disease resistance of farmed populations, and hybridization serves as an effective strategy to increase genetic diversity [9,10]. Hybridization involves mating individuals with different genotypes to produce offspring that possess a mixture of genes from both parents [11]. Based on the genetic distance between the parents, hybridization can be classified into close hybridization and distant hybridization. Distant hybridization refers to the hybridization between species with substantial genetic distance [12]. This method enables the combination of characteristics and traits from different genera or families, thereby generating new types of variation or even new species [13,14]. These variations may lead to hybrid vigor that surpasses that of the parental lines [15,16]. For instance, the crossbreeding between *Oreochromis niloticus* (♀) and *Oreochromis mossambicus* (♂) resulted in an all-female F1 hybrid generation, a phenomenon that had not been previously documented [17]. The F1 hybrid offspring obtained by crossing *Megalobrama amblycephala* (♀) with *Erythroculter mongolicus* (♂) were compared with the growth rates of their parents [18]. The hybrid yellow catfish (*Tachysurus fulvidraco* ♀ × *Pseudobagrus vachellii* ♂) exhibited enhanced adaptability in immune response [19]. Similarly, we initiated interspecific hybridization between LP and LC in 2016. This experiment successfully produced two hybrids that could survive and grow healthily. Compared with both parental species, these hybrids exhibit significant growth advantages and possess higher nutritional value [20,21,22].

Genetic diversity refers to the extent of genetic variation within the gene pool of a species, encompassing differences in genetic makeup both between distinct populations and among individuals within the same population [23]. Microsatellites, also known as Simple Sequence Repeats (SSRs), are widely present and abundant in the genomes of organisms. The flanking regions of microsatellite DNA typically consist of more conserved single-copy sequences. Based on the varying numbers of tandem repeats, microsatellite DNA exhibits length polymorphism, making it a valuable resource for molecular loci [24,25]. Currently, microsatellite DNA marker technology has emerged as a crucial tool for assessing genetic diversity within species. This approach is indispensable for evaluating germplasm resources, facilitating molecular-marker-assisted breeding, and guiding variety selection [26,27,28]. For example, 13 microsatellite loci were employed to analyze the genetic diversity of four cultured populations of *Hypophthalmichthys molitrix* in Hubei Province [29]. A total of 19 microsatellite loci were utilized to evaluate the genetic diversity of *Sinibotia superciliaris* and *Sinibotia reevesae*, as well as their reciprocal hybrid offspring. The results showed that the genetic diversity of the hybrids was intermediate between that of the two parent species [30]. Additionally, seven microsatellite loci were used to assess the genetic diversity of female *Epinephelus fuscoguttatus* and male *Epinephelus polyphekadion*, along with their hybrid offspring. The findings revealed that the genetic diversity of the hybrids exceeded that of the parents [31].

However, the genetic diversity and genetic structure of the hybrid yellow croaker and its parents have not been analyzed. In order to further investigate the impact of hybrid breeding on the genetic diversity of fish and elucidate the genetic relationships between hybrid offspring and their parental populations, we conducted a comprehensive genetic diversity analysis using 14 microsatellite loci for LPC and LCP, and compared these with their parental lines (LP and LC). At the same time, we investigated the genetic differentiation and structure among the four populations to elucidate the genetic relationships among the hybrid offspring and parental populations, as well as subpopulation differentiation. This study provides valuable insights that can inform effective management strategies for yellow croaker populations, helping to prevent inbreeding and promote gene flow between populations. Additionally, it offers a scientific foundation for future hybrid breeding and germplasm improvement efforts in yellow croakers.

## 2. Materials and Methods

### 2.1. Sample Collection and DNA Extraction

**Figure 1:**
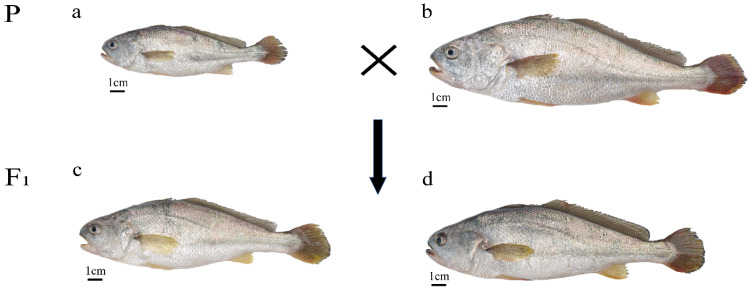
Diagram of the four yellow croaker populations. P—the parental generation; F1—the hybrid offspring; (**a**) *Larimichthys polyactis*, LP; (**b**) *Larimichthys crocea*, LC; (**c**) LP ♀ × LC ♂, LPC; (**d**) LC ♀ × LP ♂, LCP.

The experimental materials utilized in this study were sourced from Xiangshan Harbor Aquatic Seedling Co., Ltd., Ningbo, China. The small yellow croaker parent represents a family-based selected population, while the large yellow croaker parent corresponds to the novel variety “Yongdai No. 1”. Two types of hybrid offspring were produced: female small yellow croaker × male large yellow croaker (LPC) and female large yellow croaker × male small yellow croaker (LCP) (Figure 1). Genomic DNA was extracted from these samples using the Marine Animal Tissue Genomic DNA Extraction Kit (Tiangen, Beijing, China), following the manufacturer’s protocol. The purity and concentration of the extracted genomic DNA were assessed using a Protein and Nucleic Acid Quantitation Spectrophotometer (Thermo Fisher, Waltham, MA, USA). Subsequently, the DNA samples were aliquoted and diluted to a final concentration of 100 ng/μL for microsatellite analysis.

### 2.2. Microsatellite Loci Screening and Analysis

A total of 142 microsatellite loci primers for small yellow croaker and large yellow croaker were identified from the existing literature and the NCBI database [32,33,34,35,36]. PCR amplification was performed using DNA samples from four populations. The PCR reaction mixture was prepared in a total volume of 20 μL, comprising 10 μL 2×Hieff^®^ PCR Master mix (containing Hieff^®^ Taq DNA Polymerase, dNTP, MgCl_2_, Yeasen, Shanghai, China), 8 μL of ddH_2_O (Yeasen, Shanghai, China), 1 μL of DNA template, and 0.5 μL each of forward and reverse primer. A preliminary screening of 142 synthesized primers was performed using the following PCR protocol: initial denaturation at 94 °C for 5 min, followed by 35 cycles of denaturation 94 °C for 30 s, annealing at 47–63 °C for 30 s, extension at 72 °C for 30 s, and a final extension at 72 °C for 10 min. Primers were selected based on their capacity to consistently generate single, well-defined amplification bands across all four tested populations, as verified by 1.5% agarose gel electrophoresis (Novogene, Tianjin, China). This process successfully excluded primers exhibiting non-specific or weak amplification. A total of 20 primer pairs meeting the criteria were screened. Capillary electrophoresis was then employed to further evaluate the performance of these primers, and any primers displaying non-specific peaks, single-peak patterns, or poor peak resolution were excluded. Only those primers that consistently exhibited multi-peak patterns (polymorphic loci) with clear and well-resolved electropherograms were retained for subsequent analysis.

The total volume of the PCR system for the capillary electrophoresis experiment was 15 µL, comprising 7.5 µL PCR Mix (Aikerry, Yongzhou, China), 6 µL ddH₂O, 0.5 µL DNA template, and 0.25 µL of each primer (forward and reverse). The amplification protocol included an initial denaturation at 94 °C for 3 min, followed by 35 cycles of 94 °C for 20 s, 58 °C for 20 s, and 72 °C for 40 s, with a final extension at 72 °C for 5 min. Subsequently, 1 µL of the PCR product was combined with 9 µL of HiDi loading solution (ABI, Carlsbad, CA, USA). The mixture was denatured at 95 °C for 3 min and immediately placed in an ice bath. Finally, the sample was loaded onto the ABI 3730XL sequencer (ABI) for capillary electrophoresis analysis. The data were analyzed using GeneMarker V 2.2.0 software. According to the capillary electrophoresis results, a total of 14 microsatellite locus primers with high polymorphism were ultimately screened from 20 candidate primer pairs (Table 1).

### 2.3. Data Processing and Analysis

For the analysis of microsatellite loci, allele sizes were determined using GeneMarker V 2.2.0. A Micro-Checker 2.2.3 was employed to detect potential allele dropout and estimate the frequency of null alleles at each locus in R 4.4.3 [37]. Linkage disequilibrium (LD) and the corresponding *p*-values between pairs of loci were calculated using the online GenePop tool (https://genepop.curtin.edu.au, (accessed on 18 April 2025)) [38]. Subsequently, the results were visualized as a heatmap using R 4.4.3. Fundamental genetic diversity parameters, including the observed alleles number (*N_a_*), the effective alleles number (*N_e_*), the Shannon–Wiener index (*I*), observed heterozygosity (*H_o_*), and expected heterozygosity (*H_e_*), were calculated using GENALEX 6.51 b2 [39]. The polymorphism information content (*PIC*) and number of alleles (*K*) were estimated using CERVUS 3.0.7.0 [40]; the homozygosity (*H_m_*) [41] and fixation index (*F_is_*) [23] were computed using the following formulas:

(1) $$H_{m}=1-H_{o}$$

(2) $$F_{is}=\left(H_{e}-H_{o}\right)/H_{e}$$

Subsequently, one-way analysis of variance (ANOVA) was performed for each parameter value using SPSS 27.0.1 followed by Duncan’s multiple comparison test to assess pairwise differences between groups. The significance level was set at *p* < 0.05, and the post-hoc test results are presented in Table S1. Additionally, the Hardy–Weinberg equilibrium (*HWE*) test was performed for each locus using GENALEX 6.51 b2 [39] to identify the number of loci (*N_HW_*), exhibiting significant deviations in the four populations (*p* < 0.05).

The molecular variance analysis (AMOVA) and pairwise *Fst* values among the four populations were calculated using ARLEQUIN 3.5.2.2 [42] to evaluate population differentiation. Additionally, *F*-statistics were calculated, and their significance was assessed through 1000 permutations (*p* < 0.001). *Nei’s* genetic distances (*Nei’s* D) among the four populations were calculated using GENALEX 6.51 b2 [39]. Principal Component Analysis (PCoA) was performed using R 4.4.3 based on 14 loci, and a three-dimensional scatter plot was generated to visualize the results. An unbiased genetic distance matrix, based on *Nei’s* method, was calculated, and a Neighbor-Joining (NJ) phylogenetic tree was constructed from this matrix using MEGA-X 10.1.8. To further investigate the genetic structure of the four populations, Bayesian clustering analysis was conducted using STRUCTURE 2.3.4 [43] to determine the proportion of all parental genes in the genomes of each hybrid individual, in order to further assess the genetic diversity among hybrid populations. In the parameter settings, the Length of Burnin of Period was set to 15,000, followed by 150,000 MCMC replications after Burnin; K values ranged from 1 to 4, with each K value replicated 15 times. The results were visualized using StructureSelector (http://lmme.qdio.ac.cn/StructureSelector/, (accessed on 25 March 2025)) [44]. Subsequently, ΔK and Mean LnP(K) for each K value were plotted using Origin 2024.

## 3. Results

### 3.1. Genetic Diversity

According to the analysis conducted by the Micro-Checker, no significant allele loss was detected at any locus. Additionally, the frequency of null alleles across all 14 loci remained below 0.2 (Table 1), categorizing them as low-frequency null alleles. In this study, linkage disequilibrium analysis was performed on 14 microsatellite markers, resulting in a total of 91 pairwise locus combinations. As shown in Figure S1, 57 pairs of loci exhibited significant linkage equilibrium (*p* < 0.05). The remaining loci may potentially be in a state of linkage disequilibrium. Nevertheless, no locus demonstrated significant global linkage disequilibrium, suggesting that these loci exhibit good independence.

Through the analysis of genetic diversity at 14 microsatellite loci in a total of 120 individuals from four populations (LP, LC, LPC, and LCP), these 14 loci exhibited high levels of polymorphism across all four populations, because they all have multiple alleles. A total of 1361 alleles were identified, with LCP exhibiting the highest count at 419, followed by LP with 384, LPC with 309, and LC having the lowest count at 249 (Table 2). The number of alleles for LP varies from 6 (L6) to 45 (L3). For LPC, the range is from 12 (L12) to 33 (L4). In the case of LCP, it spans from 12 (L10) to 43 (L2). Lastly, for LC, the number of alleles ranges from 9 (L10) to 27 (L13). Meantime, the *PIC* values for these populations were all above 0.5, with the highest value recorded in LCP (0.94), followed by LPC (0.92), LP (0.91), and LC (0.88), resulting in an average *PIC* value of 0.91. In the comparison of other fundamental genetic diversity parameters among the four populations (Table 3), for LP, *N_a_* at the 14 loci ranged from 3 (L6) to 26 (L2), with a mean of 11.57; *N_e_* varied from 1.07 (L6) to 17.82 (L2), averaging 6.73; and *I* fluctuated between 0.17 (L6) to 3.05 (L2), with a mean of 1.88. For LC, *N_a_* ranged from 4 (L6, L10, L11 and L12) to 12 (L2), averaging 7.43; *N_e_* varied from 1.79 (L10) to 4.68 (L13), averaging 3.22; and *I* ranged from 0.82 (L10) to 1.86 (L9), with an average of 1.40. For LPC, *N_a_* ranged from 3 (L12) to 14 (L2), averaging 8.64; *N_e_* varied from 2.46 (L10) to 7.50 (L4), averaging 4.71; and *I* ranged from 1.05 (L10) to 2.24 (L2) with an average of 1.71. For LCP, *N_a_* ranged from 5 (L10) to 23 (L2), averaging 11.64; *N_e_* varied from 2.65 (L10) to 12.59 (L2), averaging 6.03; and *I* ranged from 1.11 (L10) to 2.81 (L2), with an average of 1.94.

By conducting an analysis of the average values of five parameters (*K*, *PIC*, *N_a_*, *N_e_*, *I*) for the four populations, it was revealed that LCP exhibits the highest genetic diversity (highest *PIC* value), whereas LC demonstrates the lowest genetic diversity (lowest values across all five parameters). Although LPC shows a slightly higher *PIC* value than LP, there is no statistically significant difference between these two populations. In contrast, LP significantly surpasses LPC in terms of the other four parameters. Consequently, the ranking of genetic diversity among the four populations can be summarized as LCP > LP > LPC > LC. By comparing the average value of *H_e_* in the four populations, we found that LCP was the highest, with no significant difference between LP and LPC, while LC had the lowest *H_e_* and was significantly lower than LCP. This also indicates that LCP has the highest genetic diversity, while LC has the lowest genetic diversity. In addition, the observed average value of *H_o_* in both hybrid populations exceeded that of their respective parental populations. The average value of *H_m_* and *F_is_* were higher in LP and LC compared to the two hybrid populations. Notably, the average value of *F_is_* greater than 0 in LP and LC populations suggests a potential deficiency of heterozygotes and possible inbreeding within these populations (Table 4 and Table 5). Measures such as increasing population size and optimizing breeding strategies should be implemented to reduce *F_is_* values, thereby preserving genetic diversity and promoting population health. Additionally, it was observed that LC exhibited the highest number of locus-population combinations deviating from Hardy–Weinberg equilibrium (*N_HW_* = 10), followed by LPC (*N_HW_* = 8) and LCP (*N_HW_* = 6), while LP showed the fewest deviations (*N_HW_* = 5). Moreover, we found that locus L11 demonstrated highly significant deviation in all four populations (*p* < 0.05). This indicates that the genotype frequencies of this locus do not conform to the expectations of random mating in these populations.

### 3.2. Genetic Differentiation

As illustrated in Table 6, the AMOVA analysis results revealed that the percentage of variation among populations was 3.76%; among individuals within populations, it was 21.42%; within individuals, it was 74.82%. These findings indicate that the majority of genetic variation occurs within individuals. The *F_IT_* value for the four populations was 0.252, suggesting that the observed frequency of heterozygotes was 25.2% lower than the expected value across the total population. The *F_IS_* value of 0.223 indicates significant inbreeding within the entire population. Additionally, the *F_ST_* value of 0.038 among the four populations indicates a low level of genetic differentiation, which may be attributed to frequent gene flow between populations, resulting in minimal genetic divergence.

The genetic distance among four populations, as measured by pairwise *F_st_* values, ranges from 0.019 (LPC-LCP) to 0.074 (LP-LC). The *F_st_* values between LPC and its parental populations were 0.031 for LP and 0.053 for LC, while those between LCP and its parental populations were 0.029 for LP and 0.041 for LC. *Nei’s* genetic distance among the four populations ranged from 0.357 (LPC-LCP) to 1.861 (LP-LC) (Table 7). These results are consistent with the *F_st_* values and suggest moderate levels of genetic differentiation between each population and their respective parents.

### 3.3. Population Structure

**Figure 2:**
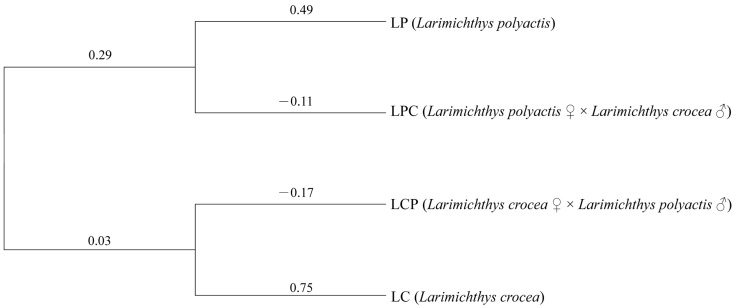
Neighbor-Joining phylogenetic tree of four yellow croaker populations constructed using *Nei’s* unbiased genetic distance based on microsatellite data. Branch values indicate pairwise genetic distance. Negative branch lengths are algorithmic artifacts of the NJ tree reconstruction process and do not reflect biological distances.

**Figure 3:**
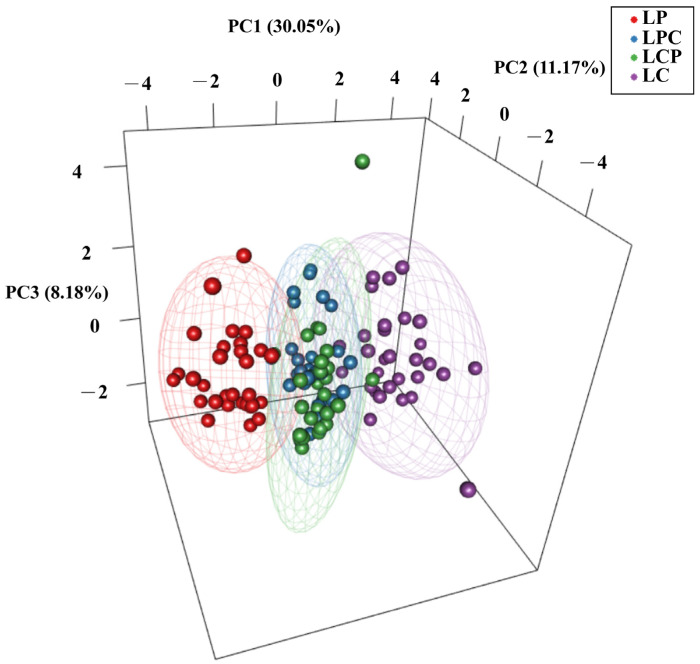
Principal Coordinates Analysis (PCoA) of four populations based on 14 microsatellite loci.

**Figure 4:**
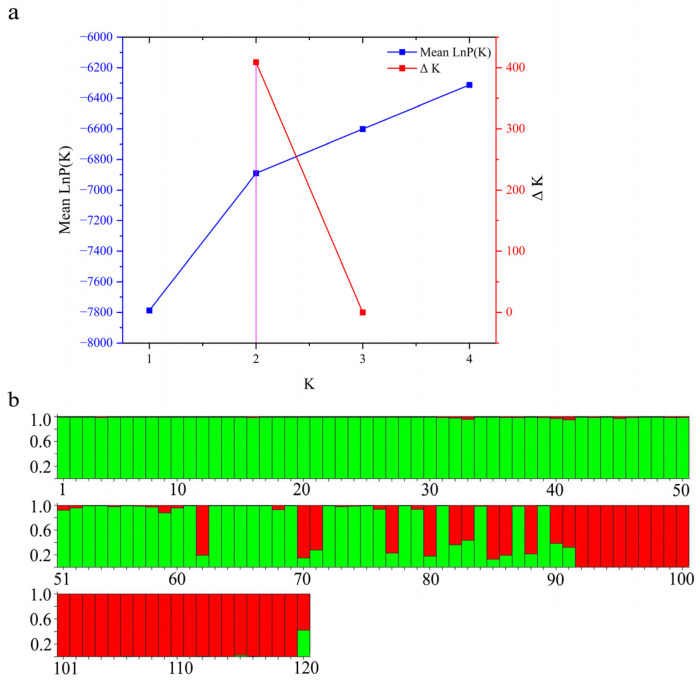
Bayesian analysis (structure) results of four yellow croaker populations based on 14 microsatellite loci. (**a**) ΔK and Mean LnP(K) values from structure analysis. The pink line in the figure serves as a vertical reference line to accurately determine the position of the K value on the horizontal axis.; (**b**) structure cluster analysis with K = 2. Each vertical column represents an individual sample, where different color segments represent distinct genetic clusters (1–30: LP; 31–60: LPC; 61–90: LCP; 91–120: LC). The numerical values on the left represent the proportion of parental genes.

To investigate the relationships among the four populations, we constructed an NJ tree using *Nei’s* unbiased genetic distance based on 14 microsatellite loci (Figure 2). The populations were divided into two main branches, with the two hybrid populations clustering closely with their respective maternal populations. Notably, the genetic distance between LCP and LC was greater than that between LPC and LP. According to the three-dimensional scatter plot, the cumulative contribution rate of the three principal components reached 49.4%. LP and LC exhibited distinct separation in three-dimensional space, suggesting substantial genetic differences at the selected SSR loci. Furthermore, partial overlap is observed between the two hybrid populations. (Figure 3). The Bayesian clustering analysis further corroborated the distinct population structure among the four populations, identifying two primary clusters (K = 2; Figure 4a), although other K values may potentially reflect secondary population structures (Figures S2 and S3). As the number of subgroups, K, increased, the average natural logarithm of the data probability Mean LnP(K) and the change in log probability ΔK were assessed (Table S2). Notably, when K = 2, the inflection point of Mean LnP(K) reached its peak value. This suggests that K = 2 is the most likely model, indicating that the four populations can be divided into two distinct subgroups. Specifically, LP and LC show significant differentiation. In the LPC population, the majority of genes are derived from LP. In contrast, within the LCP population, approximately 63% of individuals exhibit gene profiles closely resembling those in LPC, while the remaining individuals in the LCP population display a hybrid combination of genes originating from both parental lineages (Figure 4b).

## 4. Discussion

The growth performance, disease resistance, and environmental adaptability of a species are closely associated with its level of genetic diversity. Molecular markers serve as crucial tools for evaluating genetic diversity within populations. *PIC*, *Na*, *Ho*, *He*, and *I* are essential for quantifying genetic diversity detected by molecular markers. Higher values of these indicators generally reflect greater genetic diversity within the population. Specifically, higher *PIC* values indicate greater marker polymorphism within the population. In this study, 14 microsatellite loci were successfully amplified and analyzed, with *PIC* values exceeding 0.5 across all four populations, suggesting a high degree of polymorphism. For the LP and LC, the *PIC* values ranged from 0.63 to 0.97 and 0.77 to 0.94, respectively; *Na* values ranged from 3 to 26 and 4 to 12; *Ne* values ranged from 1.07 to 17.82 and 1.79 to 4.68; *I* values ranged from 0.17 to 3.05 and 0.82 to 1.86; *Ho* values ranged from 0.07 to 0.97 and 0.10 to 0.77; *He* values ranged from 0.06 to 0.94 and 0.44 to 0.79. These results are consistent with previous studies using the same loci, showing no significant differences [35,36].

We observed that the *H_o_* values for all four populations ranged from 0.54 to 0.90, the *H_e_* values varied between 0.66 and 0.79, and the *H_m_* values spanned from 0.10 to 0.46. These results suggest that the four populations examined in this study possess relatively high levels of genetic diversity. Furthermore, we noted that, for most loci of the parents, *H_e_* exceeded *H_o_*, indicating *F_is_* > 0. This suggests that these populations exhibit a certain degree of heterozygote deficiency and inbreeding [45]. To prevent genetic decline, measures such as optimizing breeding strategies and increasing population size should be implemented. The genetic diversity of the four populations can be ranked as follows: LCP > LP > LPC > LC. LCP possesses the highest level of genetic diversity, exceeding that of both parental species. This observation aligns with previous studies on brown-marbled grouper (*Epinephelus fuscoguttatus* ♀ × *Epinephelus polyphekadion* ♂) [31] and (*Epinephelus fuscoguttatus* ♀ × *Epinephelus tukula* ♂) [46]. This can be attributed to gene recombination during hybridization, where new genotypes are generated through the recombination of parental genes, thereby increasing genetic diversity [11,16]. While the genetic diversity of LPC lies between that of the two parents. This finding corroborates report on the reciprocal hybrid of the two loaches (*Sinibotia superciliaris* and *Sinibotia reevesae*), which demonstrated intermediate genetic diversity between the two parent species [30].

After evaluating the *HWE* for the four populations based on 14 loci, we found varying degrees of deviation across these populations. Notably, locus L11 exhibited highly significant deviations from *HWE* in all four populations. This suggests that the genotype frequencies at this locus do not align with the expectations under random mating within these populations, potentially due to inbreeding, insufficient sample size, or a unique mechanism specific to this locus [40]. Consequently, further investigation is warranted to elucidate the reasons behind the deviations from *HWE* at these 14 loci. Moreover, our study revealed that the genetic diversity of LCP in the hybrid population exceeds that of LPC, with fewer loci deviating from *HWE*. Thus, LCP may serve as a valuable resource for developing high-quality hybrid varieties.

The results of this study reveal that the variation rate among populations is 3.76%, representing the smallest proportion, whereas the variation within individuals accounts for the largest share at 74.82%. This finding aligns with the results reported for the hybrid loach (*Sinibotia superciliaris* and *Sinibotia reevesae*) [30]. These findings indicate a high level of allelic diversity within individuals and suggest limited differentiation among the four populations, likely due to frequent gene flow between them. Additionally, the *F_IT_* and *F_IS_* values for the four populations are significantly greater than zero (*p* < 0.001), indicating substantial inbreeding within the populations [47]. The *F_ST_* value of 0.038 among the four populations suggests minimal but statistically significant genetic differentiation (0 < *F_ST_* < 0.05). Comparing the genetic distances and degrees of differentiation among the four populations, LPC and LCP are found to be the most closely related, while LP and LC exhibit the greatest divergence. In comparisons between hybrids and their parents, LPC shows a closer relationship to LP, and similarly, LCP is more closely related to LP. However, the pairwise *F_st_* value between LCP and LP differs from that between LCP and LC by only 0.012, which can be considered negligible [48]. The NJ tree analysis reveals that the four populations can be divided into two distinct clusters, with each hybrid clustering with its respective maternal parents. This pattern is consistent with findings observed in brown-marbled grouper (*Epinephelus fuscoguttatus* ♀ × *Epinephelus tukula* ♂) [46] and hybrids of *Larimichthys crocea* (♀) and *Miichthys miiuy* (♂) [49]. Although phylogenetic analysis based on genetic distance effectively illustrates the overall differentiation pattern, it exhibits certain limitations in resolving complex hybridization relationships. Previous studies have highlighted that factors such as sample size, number of loci, locus heterozygosity, genetic distance, and clustering algorithms significantly influence the accuracy of phylogenetic tree construction [50]. Moreover, intrinsic factors like hybridization events and population bottlenecks further complicate the precision of phylogenetic inference [51].

Notably, the PCoA results revealed an overlapping genetic structure among the hybrids, which contrasts with the findings of the NJ tree. The differences between these two analytical methods, as well as the contributions of maternal and paternal genomes to the genetic architecture, may explain the discrepancies observed in the results. Specifically, the NJ tree emphasizes branch structure and is heavily influenced by allele frequencies. High frequencies of maternal-specific alleles at microsatellite loci can lead to tighter clustering of maternal hybrids during genetic distance calculations. In contrast, PCoA evaluates overall genetic similarity across multidimensional space. If paternal alleles are evenly distributed across multiple loci, the infiltration of paternal genes reduces nuclear divergence, causing hybrids to cluster more closely within the overall genetic structure. This decoupling between maternal lineage signals (NJ tree) and nuclear admixture (PCoA) aligns with hybridization systems where cytoplasmic and nuclear introgression rates differ [52].

In the analysis of genetic structure, the significant disparity in the proportion of maternal inheritance observed in the LPC and LCP hybrid populations (where LPC is closer to the maternal LP, and the maternal LC contribution in LCP is less than 50%) may result from a combination of the following factors: (1) nuclear–cytoplasmic interactions—in LPC hybrids, the cytoplasmic genome provided by the maternal parent (LP) may exhibit co-adaptation with the nuclear genome, leading to an increased likelihood of offspring retaining maternal nuclear genes to preserve nuclear–cytoplasmic interactions [53]; (2) selective filtering of gene introgression—in the LCP hybrid population, alleles originating from the maternal parent (LC) may be subject to negative selection in hybrid offspring, thereby causing a deviation in genomic proportions [54]. Furthermore, while genetic differentiation between the parent species and hybrids is a predictable consequence of hybridization events. The primary contribution of this study is in its clarification of how hybridization directionality, driven by maternal selection, influences genetic composition and confers a genomic stability advantage to LCP over LPC, highlighting its potential as a superior breeding material. These insights transcend mere confirmation of differentiation and offer a robust theoretical foundation for designing hybridization-based breeding strategies.

## 5. Conclusions

In this study, we investigated the genetic diversity and structure of large yellow croaker, small yellow croaker, and their reciprocal hybrid populations using 14 microsatellite loci. The results revealed that genetic diversity follows the order LCP > LP > LPC > LC, indicating that different hybrid combinations significantly influence the genetic diversity of offspring. The high genetic diversity observed in the LCP population may enhance its adaptability and evolutionary potential, making it a valuable germplasm resource with significant potential. Its genome integrates genetic variations from both parental species, serving as an intermediate breeding material for subsequent genetic improvement. Through molecular-marker-assisted selection and directional backcrossing, specific traits can be identified and introduced from hybrids into parental populations, thereby achieving germplasm innovation for target traits. We recognize that the fertility of hybrids poses a risk of genetic introgression into wild populations. To mitigate this concern, developing completely sterile hybrids remains an urgent priority requiring immediate attention. Furthermore, during the breeding process, the environment for hybrid fish should be strictly controlled to prevent escapes and minimize genetic contamination of wild populations.

## Figures and Tables

**Table 1:** Characterization of 14 microsatellite loci utilized in this study.

Locus	Reference	Repeat Motif	Primer Sequence (5′–3′)	Tm/°C	Allele Size	Fluorescent Labeling	Null Allele Frequency
**L1**	Xie et al. 2020 [36]	(TGAT)5	F: TGTAGATCGGATGCCAGTTG R: TTCATGAAACATGCAGAGGG	55	231~271	FAM	0.04112
**L2**	Xie et al. 2020 [36]	(ATAG)12	F: GGCAGCGGTGACATTATTCT R: AACTCACCGCAGAAACTGAAA	56	261~352	HEX	0.00008
**L3**	Xie et al. 2020 [36]	(AGAT)9	F: CACAGCCCACTGATGATGTC R: ATCCTCCCCCATACAAGTCC	55	274~415	FAM	−0.01262
**L4**	Wu et al. 2021 [35]	(ACAT)9	F: CACAGCCTTTCTTTGGAATCA R: CACTGTCACTTTTGCTGTATGGA	56	176~252	HEX	−0.01312
**L5**	Xie et al. 2020 [36]	(GACA)5	F: TTAGGCGATCACCAAAGTCA R: TTCAGTTTTCTGCTGGTTTCTG	55	235~249	HEX	0.01281
**L6**	Xie et al. 2020 [36]	(CCTG)7	F: AAACTCACGACCGGAACAAC R: TGTAGCTGAACGCTCATTGG	56	239~263	HEX	−0.09336
**L7**	EF635869	(TC)9(CTT)6	F: CATCTCCCCCACTCATATCG R: TTCAGACTGCTGCCCTGTC	56	281~304	FAM	−0.04372
**L8**	Xie et al. 2020 [36]	(TATT)5	F: CAATTCAAACACCGTCCTGA R: GTTTCCTGTGAATCGCCTGT	55	254~288	HEX	−0.01393
**L9**	EF635876	(CT)8	F: CTTTGCTGTGAGGCTTTTCC R: TCGCAGACAGAATCTCCAAG	57	213~264	HEX	0.08007
**L10**	KF805068	(AG)11	F: CTTCAACATTTCCTCCATTT R: GTGTTCAGGACTGCGTATTT	52	152~166	HEX	0.00609
**L11**	HQ678309	(AG)6	F: AGCCTACAGGTGAATGAGTG R: GCTTGGGTCTGAGGTTGC	55	209~256	HEX	0.05032
**L12**	Xie et al. 2020 [36]	(TGAA)5	F: ATAGCTGTCTCCATGCCCAC R: AAAATTGACCTCCAGCCAAA	55	215~235	HEX	0.12789
**L13**	KC773866	(TG)11	F: AAAGCCTCCGTCAAGCAC R: CGTATTCAAACCAGCACA	53	175~203	FAM	−0.03206
**L14**	EF635877	(CTT)6	F: CCTCCTCACCTGCTAACT R: AACAAACGAAGCCCAACT	53	353~402	HEX	−0.05425

**Table 2:** The value of *K* and *PIC* for the four populations based on 14 microsatellite loci.

Locus	*K*				*PIC*			
	LP	LPC	LCP	LC	LP	LPC	LCP	LC
**L1**	28	21	28	17	0.94	0.91	0.95	0.88
**L2**	40	29	43	23	0.97	0.95	0.97	0.86
**L3**	45	29	40	18	0.97	0.95	0.96	0.90
**L4**	36	33	41	23	0.96	0.95	0.97	0.92
**L5**	13	22	23	18	0.82	0.94	0.94	0.89
**L6**	6	13	21	12	0.63	0.85	0.93	0.86
**L7**	33	21	34	20	0.96	0.91	0.95	0.91
**L8**	34	19	29	12	0.96	0.92	0.93	0.84
**L9**	25	21	35	17	0.94	0.92	0.96	0.90
**L10**	18	15	12	9	0.90	0.89	0.85	0.77
**L11**	28	22	30	13	0.95	0.93	0.95	0.85
**L12**	21	12	23	17	0.92	0.88	0.93	0.90
**L13**	29	27	30	27	0.95	0.94	0.95	0.94
**L14**	28	25	30	23	0.94	0.94	0.96	0.90
**Total**	384	309	419	249	-	-	-	-
**Mean**	27.43	22.07	29.93	17.79	0.91	0.92	0.94	0.88

*K*, the number of alleles; *PIC*, polymorphism information content.

**Table 3:** The value of *N_a_*, *N_e_*, and *I* for the four populations based on 14 microsatellite loci.

Locus	*N_a_*				*N_e_*				*I*			
	LP	LPC	LCP	LC	LP	LPC	LCP	LC	LP	LPC	LCP	LC
**L1**	13	8	10	8	4.32	2.64	5.13	3.61	1.85	1.39	1.83	1.53
**L2**	26	14	23	12	17.82	7.35	12.59	2.77	3.05	2.24	2.81	1.61
**L3**	22	10.	17	10	16.22	6.32	8.22	4.57	2.91	2.06	2.45	1.80
**L4**	14	13	18	10	7.89	7.50	10.71	3.61	2.30	2.22	2.60	1.72
**L5**	7	10	7	6	2.25	6.32	5.23	2.85	1.16	2.09	1.77	1.26
**L6**	3	5	7	4	1.07	3.06	3.10	2.13	0.17	1.33	1.38	0.97
**L7**	11	10	13	10	7.23	4.33	5.33	4.44	2.13	1.79	2.06	1.82
**L8**	11	7	10	5	6.57	2.82	2.80	2.52	2.11	1.36	1.51	1.13
**L9**	11	9	12	10	4.75	4.47	5.75	4.60	1.88	1.71	2.04	1.86
**L10**	6	4	5	4	2.20	2.46	2.65	1.79	1.15	1.05	1.11	0.82
**L11**	9	9	9	4	5.47	3.79	4.63	2.03	1.88	1.61	1.79	0.85
**L12**	5	3	7	4	3.24	2.87	3.46	2.48	1.35	1.08	1.41	1.04
**L13**	13	10	13	9	8.87	6.02	6.98	4.68	2.34	1.98	2.13	1.77
**L14**	11	9	12	8	6.34	6.04	7.83	2.94	2.03	1.98	2.23	1.40
**Mean**	11.57 ^b^	8.64 ^ab^	11.64 ^b^	7.43 ^a^	6.73 ^b^	4.71 ^ab^	6.03 ^b^	3.22 ^a^	1.88 ^b^	1.71 ^ab^	1.94 ^b^	1.40 ^a^

*N_a_*, observed alleles number; *N_e_*, effective alleles number; *I*, Shannon–Wiener index. Different letters in the same row indicate significant differences among populations (*p* < 0.05).

**Table 4:** The value of *H_o_*, *H_e_*, and *H_m_* for the four populations based on 14 microsatellite loci.

Locus	*H_o_*				*H_e_*				*H_m_*			
	LP	LPC	LCP	LC	LP	LPC	LCP	LC	LP	LPC	LCP	LC
**L1**	0.67	0.67	0.73	0.57	0.77	0.62	0.81	0.72	0.33	0.33	0.27	0.43
**L2**	0.70	1.00	1.00	0.67	0.94	0.86	0.92	0.64	0.30	0.00	0.00	0.33
**L3**	0.97	0.93	0.97	0.67	0.94	0.84	0.88	0.78	0.03	0.07	0.03	0.33
**L4**	0.83	1.00	0.97	0.67	0.87	0.87	0.91	0.72	0.17	0.00	0.03	0.33
**L5**	0.33	0.90	0.90	0.63	0.56	0.84	0.81	0.65	0.67	0.10	0.10	0.37
**L6**	0.07	1.00	1.00	0.43	0.06	0.67	0.68	0.53	0.93	0.00	0.00	0.57
**L7**	0.80	0.97	1.00	0.77	0.86	0.77	0.81	0.78	0.20	0.03	0.00	0.23
**L8**	0.73	0.87	0.87	0.37	0.85	0.65	0.64	0.60	0.27	0.13	0.13	0.63
**L9**	0.77	0.67	0.90	0.27	0.79	0.78	0.83	0.78	0.23	0.33	0.10	0.73
**L10**	0.47	0.73	0.87	0.10	0.55	0.59	0.62	0.44	0.53	0.27	0.13	0.90
**L11**	0.47	0.63	0.90	0.50	0.82	0.74	0.78	0.51	0.53	0.37	0.10	0.50
**L12**	0.23	0.53	0.60	0.43	0.69	0.65	0.71	0.60	0.77	0.47	0.40	0.57
**L13**	0.87	1.00	0.97	0.77	0.89	0.83	0.86	0.79	0.13	0.00	0.03	0.23
**L14**	0.87	1.00	1.00	0.73	0.84	0.83	0.87	0.66	0.13	0.00	0.00	0.27
**Mean**	0.63 ^a^	0.85 ^b^	0.90 ^b^	0.54 ^a^	0.74 ^ab^	0.75 ^ab^	0.79 ^b^	0.66 ^a^	0.37 ^b^	0.15 ^a^	0.10 ^a^	0.46 ^b^

*H_o_*, observed heterozygosity; *H_e_*, expected heterozygosity; *H_m_*, Homozygosity. Different letters in the same row indicate significant differences among populations (*p* < 0.05).

**Table 5:** Chi-square test *p*-values of Hardy–Weinberg equilibrium (*HWE*) and value of *F_is_* for the four populations based on 14 microsatellite loci.

Locus	*F_is_*				*HWE*			
	LP	LPC	LCP	LC	LP	LPC	LCP	LC
**L1**	0.13	−0.07	0.09	0.22	0.184 ^NS^	0.959 ^NS^	0.039 *	0.365 ^NS^
**L2**	0.26	−0.16	−0.09	−0.04	0.000 ***	0.001 ***	0.708 ^NS^	0.655 ^NS^
**L3**	−0.03	−0.11	−0.10	0.15	0.712 ^NS^	0.028 *	0.984 ^NS^	0.000 ***
**L4**	0.05	−0.15	−0.07	0.08	0.489 ^NS^	0.000 ***	0.816 ^NS^	0.001 ***
**L5**	0.40	−0.07	−0.11	0.02	0.043 *	0.063 ^NS^	0.001 ***	0.002 **
**L6**	−0.03	−0.49	−0.48	0.18	0.998 ^NS^	0.001 ***	0.000 ***	0.058 ^NS^
**L7**	0.07	−0.26	−0.23	0.01	0.985 ^NS^	0.059 ^NS^	0.777 ^NS^	0.025 *
**L8**	0.13	−0.34	−0.35	0.39	0.003 **	0.349 ^NS^	0.946 ^NS^	0.000 ***
**L9**	0.03	0.14	−0.09	0.66	0.828 ^NS^	0.436 ^NS^	0.092 ^NS^	0.000 ***
**L10**	0.14	−0.24	−0.39	0.77	0.286 ^NS^	0.000 ***	0.000 ***	0.000 ***
**L11**	0.43	0.14	−0.15	0.01	0.000 ***	0.000 ***	0.000 ***	0.000 ***
**L12**	0.66	0.18	0.16	0.27	0.000 ***	0.000 ***	0.880 ^NS^	0.019 *
**L13**	0.02	−0.20	−0.13	0.02	0.905 ^NS^	0.354 ^NS^	0.199 ^NS^	0.603 ^NS^
**L14**	−0.03	−0.20	−0.15	−0.11	0.117 ^NS^	0.000 ***	0.036 *	0.002 **
***N_HW_***	-	-	-	-	5	8	6	10
**Mean**	0.16	−0.13	−0.15	0.19	-	-	-	-

*F_is_*, fixation index; *HWE*, Chi-square test *p*-values of Hardy–Weinberg equilibrium; *NHW*, the number of loci deviating from *HWE*. NS was not significant; * *p* < 0.05; ** *p* < 0.01; *** *p* < 0.001.

**Table 6:** AMOVA analysis of the four populations based on 14 microsatellite loci.

Source of Variation	*d.f.*	Source of Squares	Variance Components	Percentage of Variation (%)	*F*-Statistic
**Among populations**	3	70.387	0.25705	3.76	*F_IS_* = 0.223 *** F_ST_ = 0.038 *** F_IT_ = 0.252 ***
**Among Individuals/** **within populations**	116	932.550	1.46336	21.42	
**Within individuals**	120	613.500	5.11250	74.82	
**Total**	239	1616.438	6.83292	100	

*F_IS_*, inbreeding coefficient within individuals, *F_ST_*, fixation index between subpopulations, *F_IT_*, inbreeding coefficient within subpopulations. *** *p* < 0.001.

**Table 7:** The pairwise *F_st_* and *Nei’s D* values of the four populations based on 14 microsatellite loci.

	LP	LPC	LCP	LC
**LP**		0.430	0.440	1.861
**LPC**	0.031 ***		0.357	0.729
**LCP**	0.029 ***	0.019 ***		0.633
**LC**	0.074 ***	0.053 ***	0.041 ***	

The values below the diagonal are pairwise *F_st_*, the values above the diagonal are *Nei’s D*. *** *p* < 0.001.

## Data Availability

The original contributions presented in this study are included in the article. Further inquiries can be directed to the corresponding authors.

## Supplementary Materials

The following supporting information can be downloaded at: https://www.mdpi.com/article/10.3390/ani15101360/s1, Table S1: Duncan’s multiple range test results for genetic diversity parameters across four populations. Table S2: Results of Bayesian genetic structure analysis. Figure S1: Heatmap illustrating linkage disequilibrium between pairs of loci based on 14 microsatellite loci. Below the diagonal are indicators showing the statistical significance of the linkage disequilibrium. Figure S2: Structure cluster analysis with K = 3. Each vertical column represents an individual sample, where different color segments represent distinct genetic clusters (1–30: LP; 31–60: LPC; 61–90: LCP; 91–120: LC). Figure S3: Structure cluster analysis with K = 4. Each vertical column represents an individual sample, where different color segments represent distinct genetic clusters (1–30: LP; 31–60: LPC; 61–90: LCP; 91–120: LC).
