# Supplementary material for: Comparative Analysis of Genetic Structure and Diversity in Larimichthys polyactis, Larimichthys crocea, and Their Reciprocal Hybrids Based on Microsatellite Loci
Source: Animals (Basel). 2025 May 8;15(10):1360. doi: 10.3390/ani15101360 (PMC12108211; doi:10.3390/ani15101360)
Supplement: Supplementary file 1 [file animals-15-01360-s001.zip › Table S1.pdf]

Table S1. Duncan's multiple range test results for genetic diversity parameters across four populations.

Population	N_a		N_e		I		H_o		H_e		H_m	
	a	b	a	b	a	b	a	b	a	b	a	b
**LP**		11.57		6.73		1.88	0.63		0.74	0.74		0.37
**LPC**	8.64	8.64	4.71	4.71	1.71	1.71		0.85	0.75	0.75	0.15	
**LCP**		11.64		6.03		1.94		0.90		0.79	0.10	
**LC**	7.43		3.22		1.40		0.54		0.66			0.46

Na, observed alleles number; *Ne*, effective alleles number; *I*, Shannon-Wiener index; *Ho*, observed heterozygosity; *He*, expected heterozygosity; *Hm*, Homozygosity; a and b indicate significant difference ($P < 0.05$).
