# Supplementary material for: Comparative Analysis of Genetic Structure and Diversity in Larimichthys polyactis, Larimichthys crocea, and Their Reciprocal Hybrids Based on Microsatellite Loci
Source: Animals (Basel). 2025 May 8;15(10):1360. doi: 10.3390/ani15101360 (PMC12108211; doi:10.3390/ani15101360)
Supplement: Supplementary file 1 [file animals-15-01360-s001.zip › Table S2.pdf]

Table S2. Results of Bayesian genetic structure analysis.

K	Reps	Mean LnP(K)	Stdev LnP(K)	ΔK
**1**	15	-7787.03333	0.9737	NA
**2**	15	-6890.68667	1.48414	408.81633
**3**	15	-6601.08	26.48946	0.04228
**4**	15	-6312.59333	66.73972	NA
