# Supplementary figures and images for: Comparative Analysis of Genetic Structure and Diversity in Larimichthys polyactis, Larimichthys crocea, and Their Reciprocal Hybrids Based on Microsatellite Loci
Source: Animals (Basel). 2025 May 8;15(10):1360. doi: 10.3390/ani15101360 (PMC12108211; doi:10.3390/ani15101360)

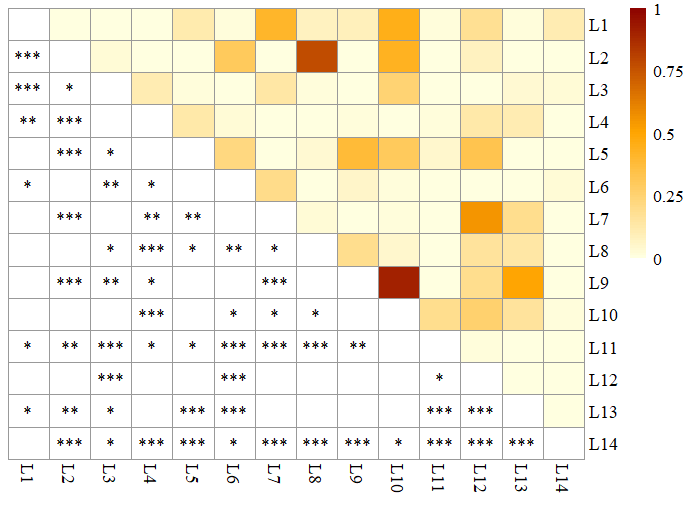

Supplement: Supplementary file 1 [file animals-15-01360-s001.zip › Figure S1.tif]

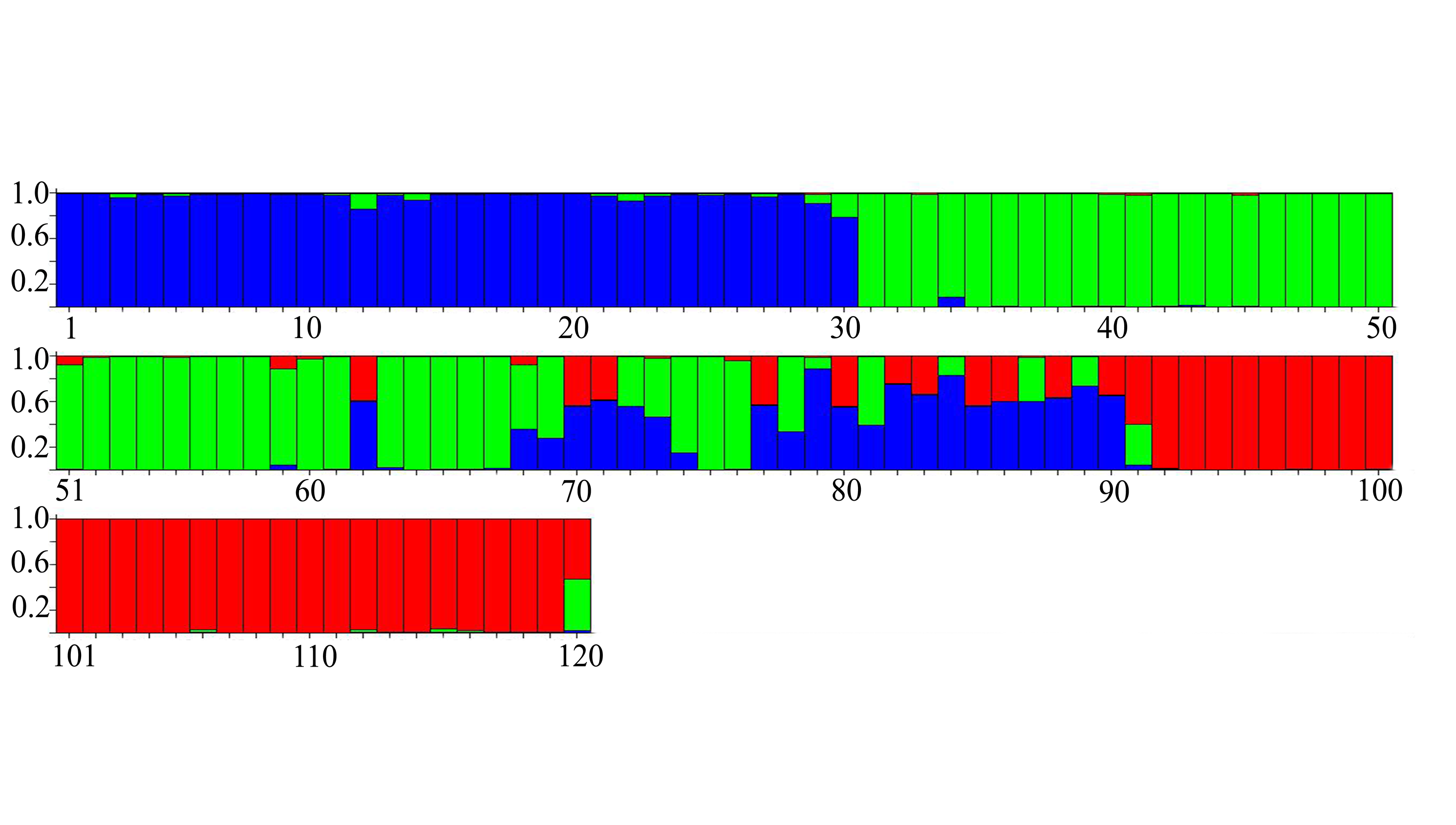

Supplement: Supplementary file 1 [file animals-15-01360-s001.zip › Figure S2.tif]

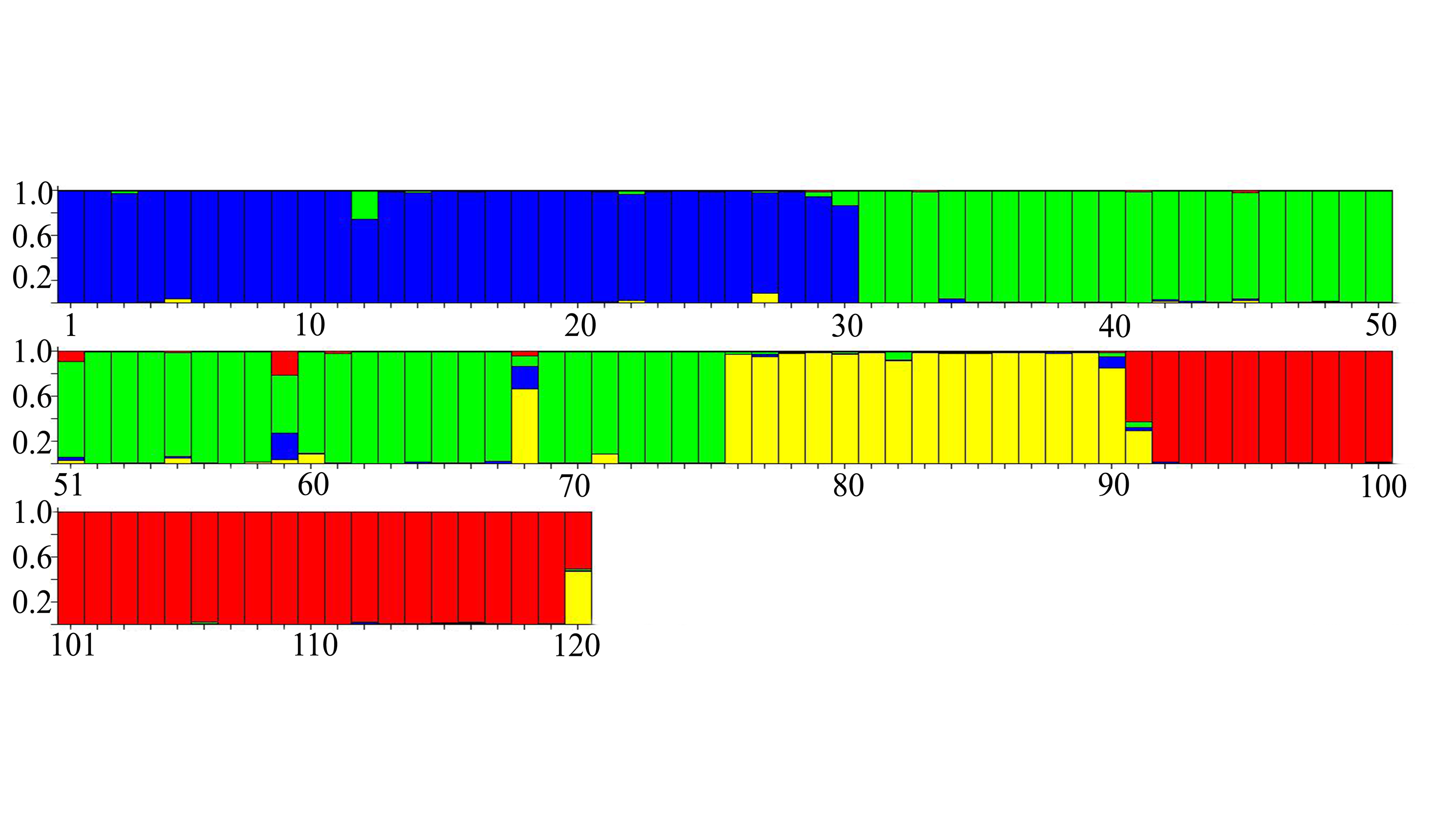

Supplement: Supplementary file 1 [file animals-15-01360-s001.zip › Figure S3.tif]
